# Supplementary material for: The CXCR4–CXCL12-Axis Is of Prognostic Relevance in DLBCL and Its Antagonists Exert Pro-Apoptotic Effects In Vitro
Source: Int J Mol Sci. 2019 Sep 24;20(19):4740. doi: 10.3390/ijms20194740 (PMC6801866; doi:10.3390/ijms20194740)
Supplement: Supplementary file 1 [file ijms-20-04740-s001.pdf]

Supplementary Material

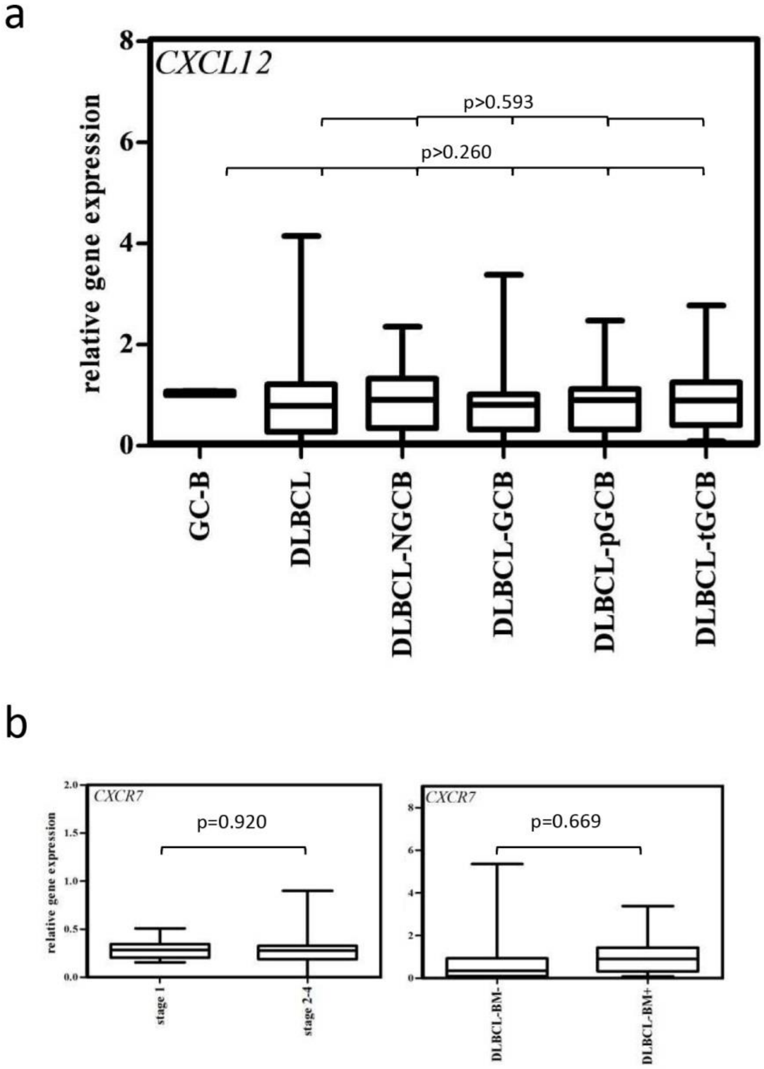

Figure S1. *CXCR7* expression in DLBCL: (**a**) Expression analysis of *CXCR7* in non-neoplastic control germinal center B cells (GC-B) and diffuse large B cell lymphoma cells (DLBCL) consisting of DLBCL-NGCB and DLCBL-GCB by RQ-PCR. GCB-DLBCL were further subdivided into primary (DLBCL-pGCB) and transformed DLBCL (DLBCL-pGCB) originating from follicular lymphoma. (**b**) Expression analysis of *CXCR7* in DLBCL samples with early (stage 1) and advanced stage (stage 2–4) (left graph) and DLBCL samples with and without bone marrow infiltration (right graph) by RQ-PCR. mRNA expression levels were calculated as the relative expression in comparison to GC-B cells. The comparison of the expression levels was performed by using the Mann–Whitney U-test or Student's t-test.

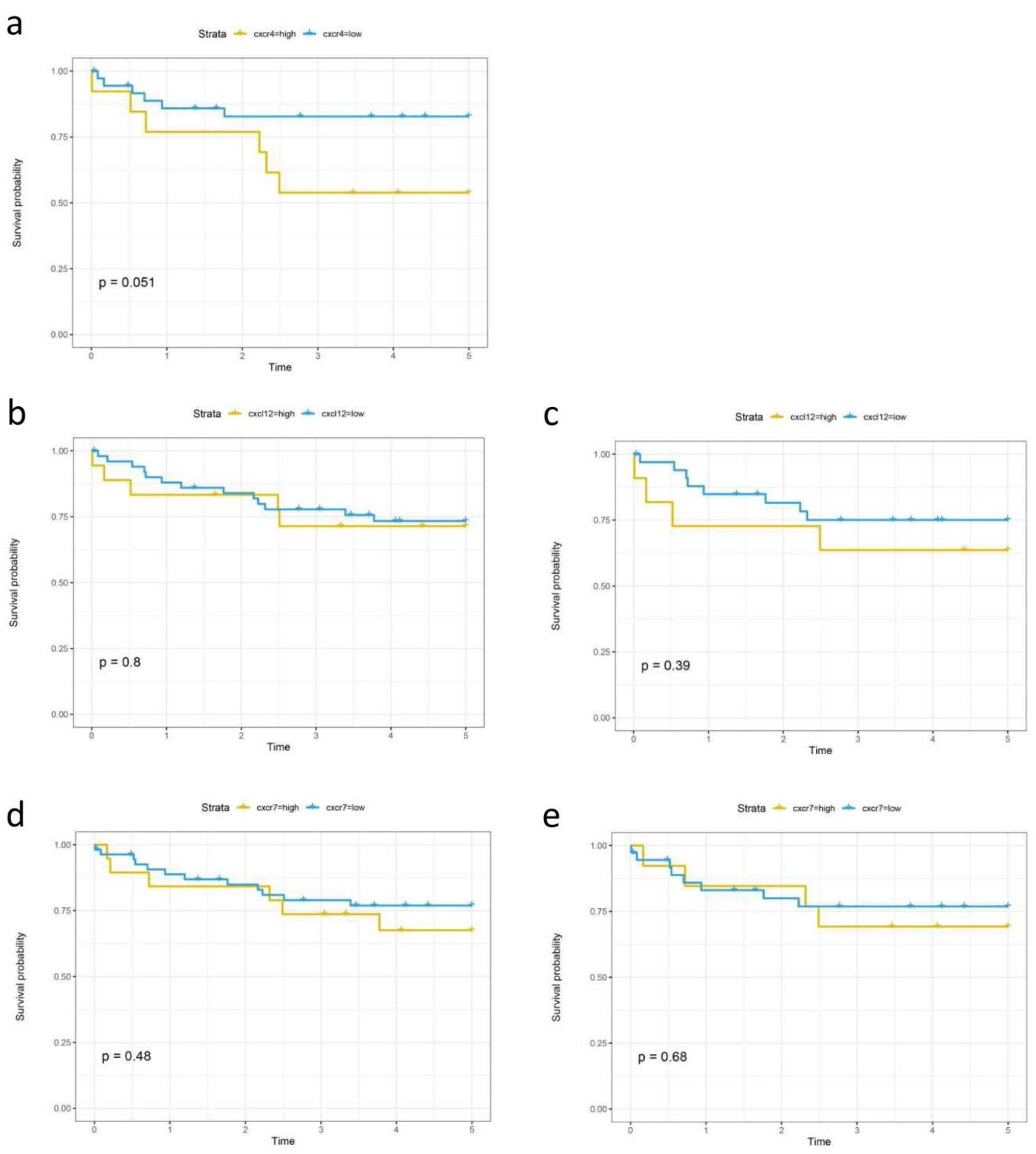

Figure S2. Survival analysis based on expression levels of *CXCR4*, *CXCL12*, and *CXCR7*. (**a**) Probability of 5-year-survival in de novo DLBCL patients based on CXCR4 expression. (**b**,**c**) Probability of 5-year-survival in all (**b**) and de novo (**c**) DLBCL patients based on *CXCL12* expression. (**d**,**e**) Probability of 5-year-survival in all (**d**) and de novo (**e**) DLBCL patients based on *CXCR7* expression.

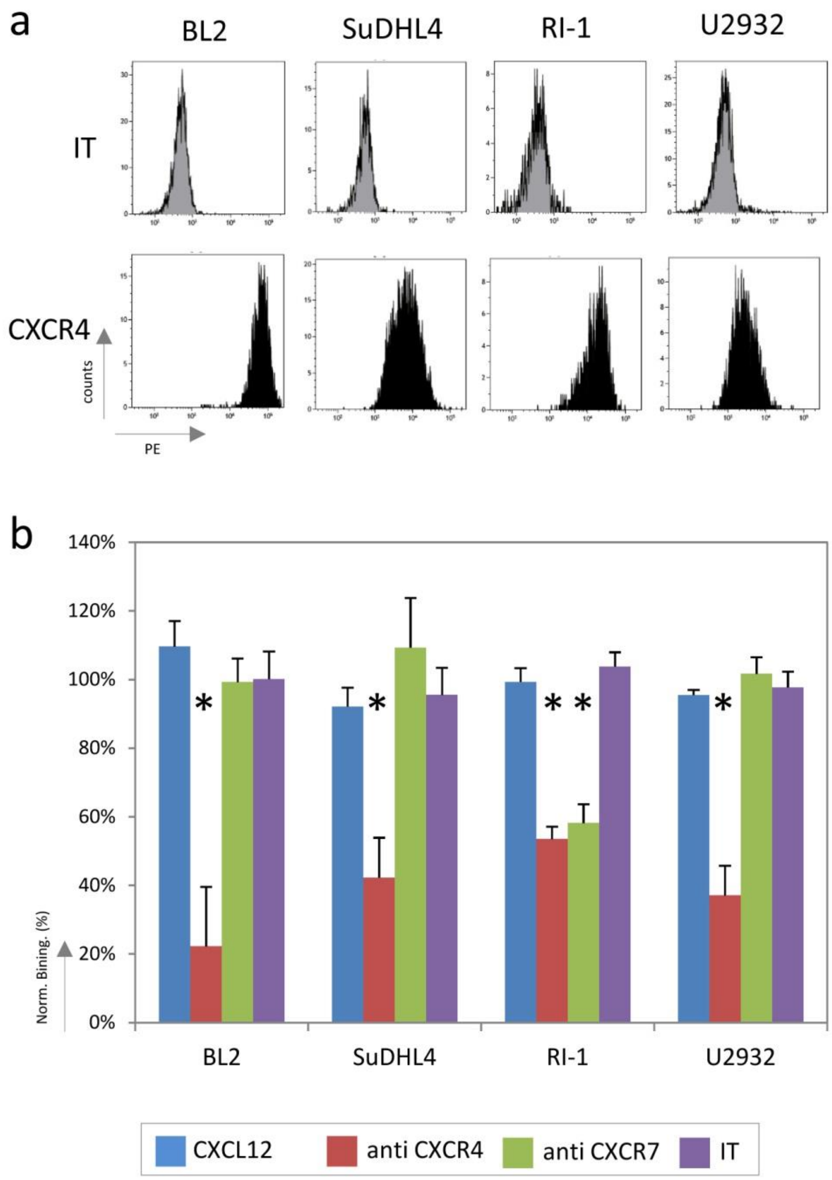

Figure S3. CXCR4 expression and CXCL12^{AF647} binding: (**a**) Surface expression of CXCR4 on SuDHL4, RI-1, U2932, and BL2 cell lines as determined by flow cytometry using a Phycoerythrin (PE) labeled antibody targeting CXCR4 (clone 2B11). (**b**) Percentage of CXCL12^{AF647} binding in the presence and absence of blocking antibodies against CXCR4 and CXCR7 as determined by flow cytometry. CXCL12 is bound by CXCR4 in all cell lines examined, while it is also bound by CXCR7 on RI-1 cells. IT denotes isotype control. * indicates significant reduction of CXCL12^{AF647} binding in the presence of antibodies against CXCR4 or CXCR7 compared to the binding isotype controls ($p < 0.01$).

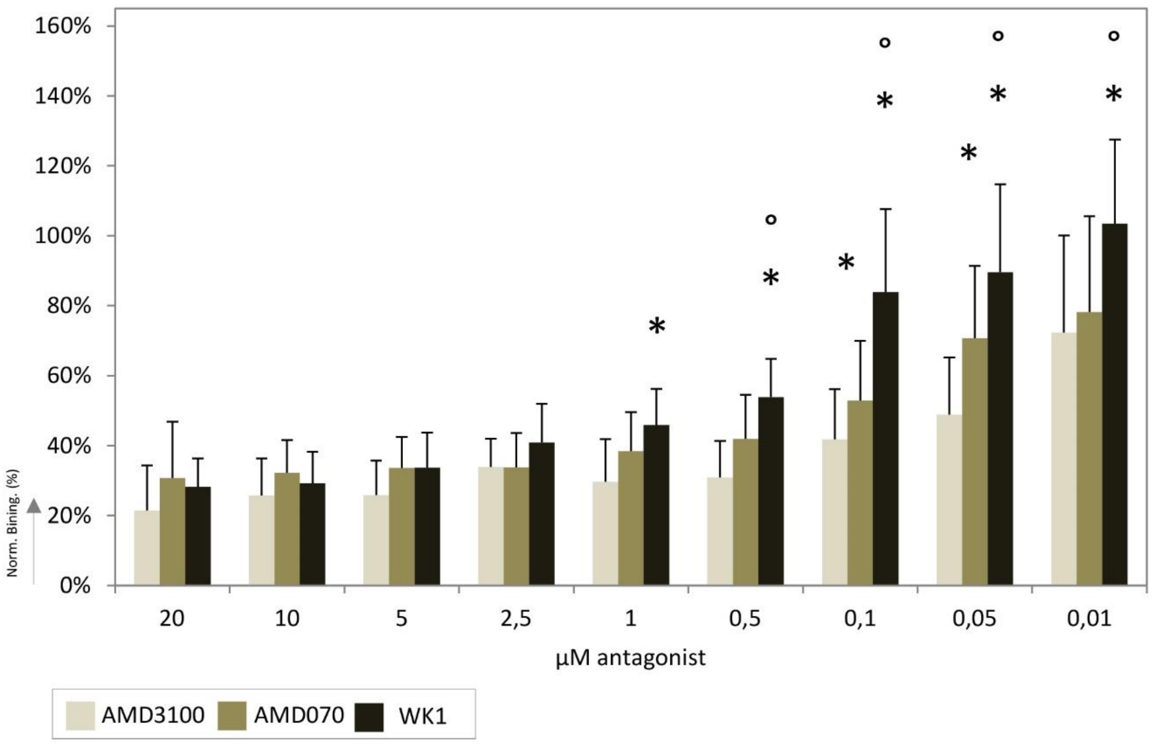

Figure S4. CXCR4 antagonists and CXCL12^AF647 binding. Percentage of CXCL12^AF647 binding in the presence of AMD3100, AMD070, and its niacin derivative WK1 on BL2 cells. CXCL12^AF647 binding to CXCR4 is inhibited by all three antagonists tested. * indicates significant differences of CXCL12^AF647 binding in the presence of AMD070 and WK1, respectively, in comparison to AMD3100 ($p < 0.01$). ° indicates significant differences of CXCL12^AF647 binding in the presence of WK1 in comparison to AMD070 ($p < 0.05$).

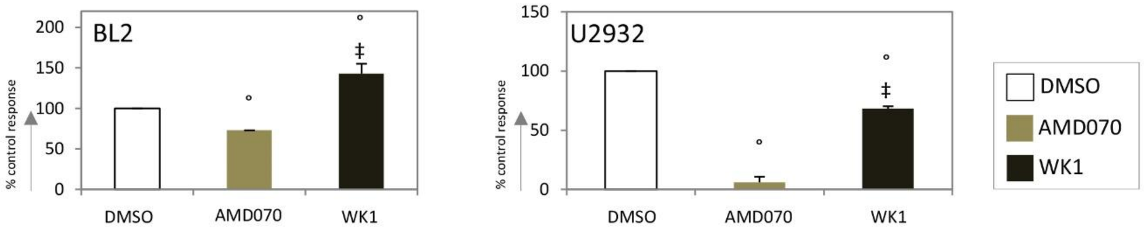

Figure S5. CXCR4 antagonists and transmigration. Percentage of transmigration of BL2 (**left**) and U2932 (**right**) cells in the presence of AMD070 and its niacin derivative WK1, respectively, in comparison to transmigration with DMSO as control. Transmigration was inhibited in both cell lines using AMD070 while inhibition by WK1 was not that efficient. ° indicates significant inhibition of transmigration compared to the DMSO control ($p < 0.05$). ‡ indicates significantly lower inhibition of WK1 compared to AMD070 ($p < 0.05$).

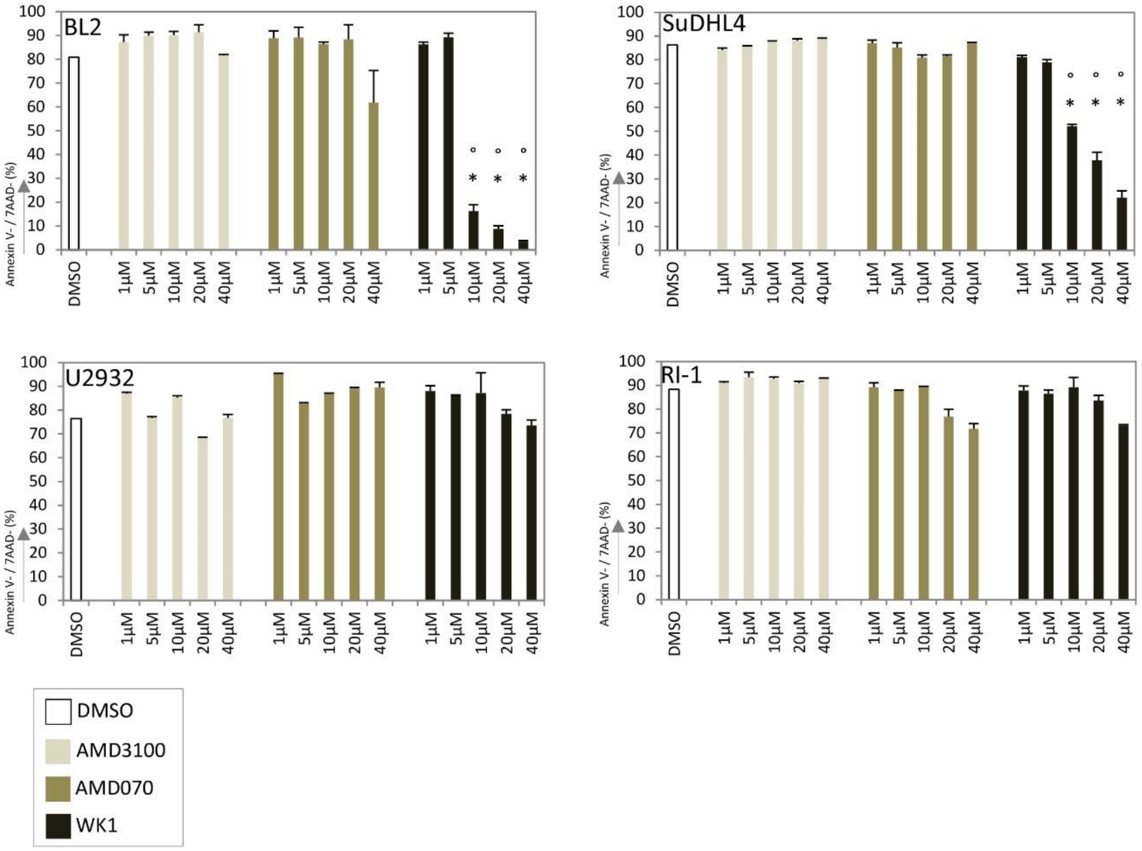

Figure S6. CXCR4 antagonists and cell viability. Percentage of Annexin V/ 7AAD positive SuDHL4, RI-1, U2932 and BL2 cell lines in the presence of increasing concentrations of AMD3100, AMD070, and its niacin derivative WK1 as determined by flow cytometry and compared to DMSO treated control cells. Only treatment of BL2 and SUDHL4 cells with 10 µM, 20 µM, and 40 µM WK1 led to a decrease in viable, Annexin V/ 7AAD negative cells, while all other cell lines or antagonists showed no effect. * indicates significantly reduced viability compared to AMD3100 ($p < 0.05$). ° indicates significantly reduced viability compared to AMD070 ($p < 0.05$).

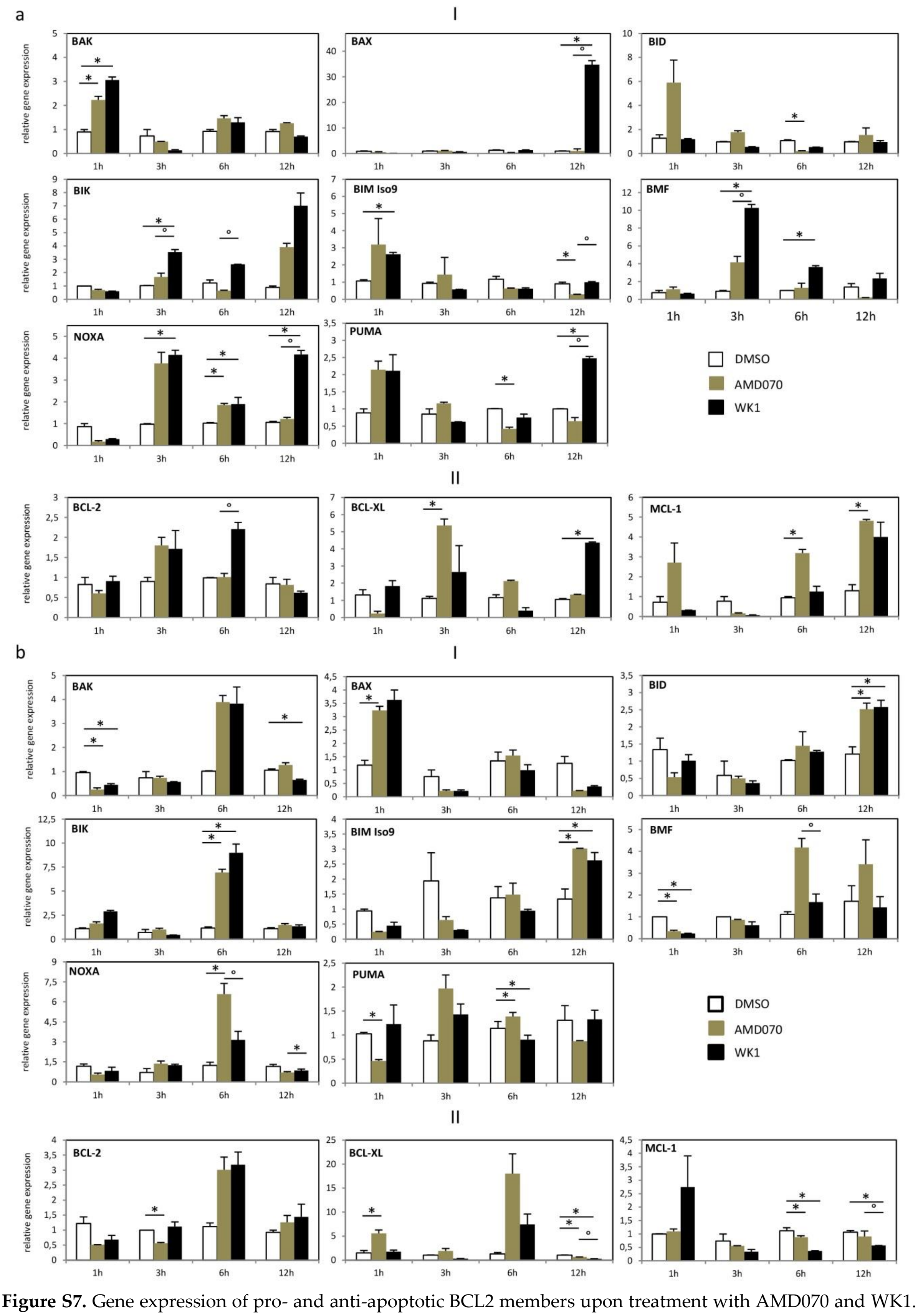

Figure S7. Gene expression of pro- and anti-apoptotic BCL2 members upon treatment with AMD070 and WK1. (**a**) BL2 (as Burkitt model) and (**b**) U2932 (as NGCB-DLBCL model) were treated with 40μM AMD070 or its niacin derivative WK1 and after 1 h, 3 h, 6 h, and 12 h gene expression levels of (I) pro-apoptotic and (II) anti-apoptotic BCL2 family members were determined in comparison to the DMSO treated control cells by RQ PCR. mRNA expression levels were calculated as the relative expression in comparison to DMSO controls. Each bar represents the mean values of expression levels ± standard error of the mean (SEM). The comparison of the expression levels

was performed by using the Mann–Whitney U-test or Student's t-test. * Expression is significant to the DMSO control ($p \leq 0.05$). ° Expression is significantly different compared to the AMD070 and WK1 treatment ($p \leq 0{,}05$).

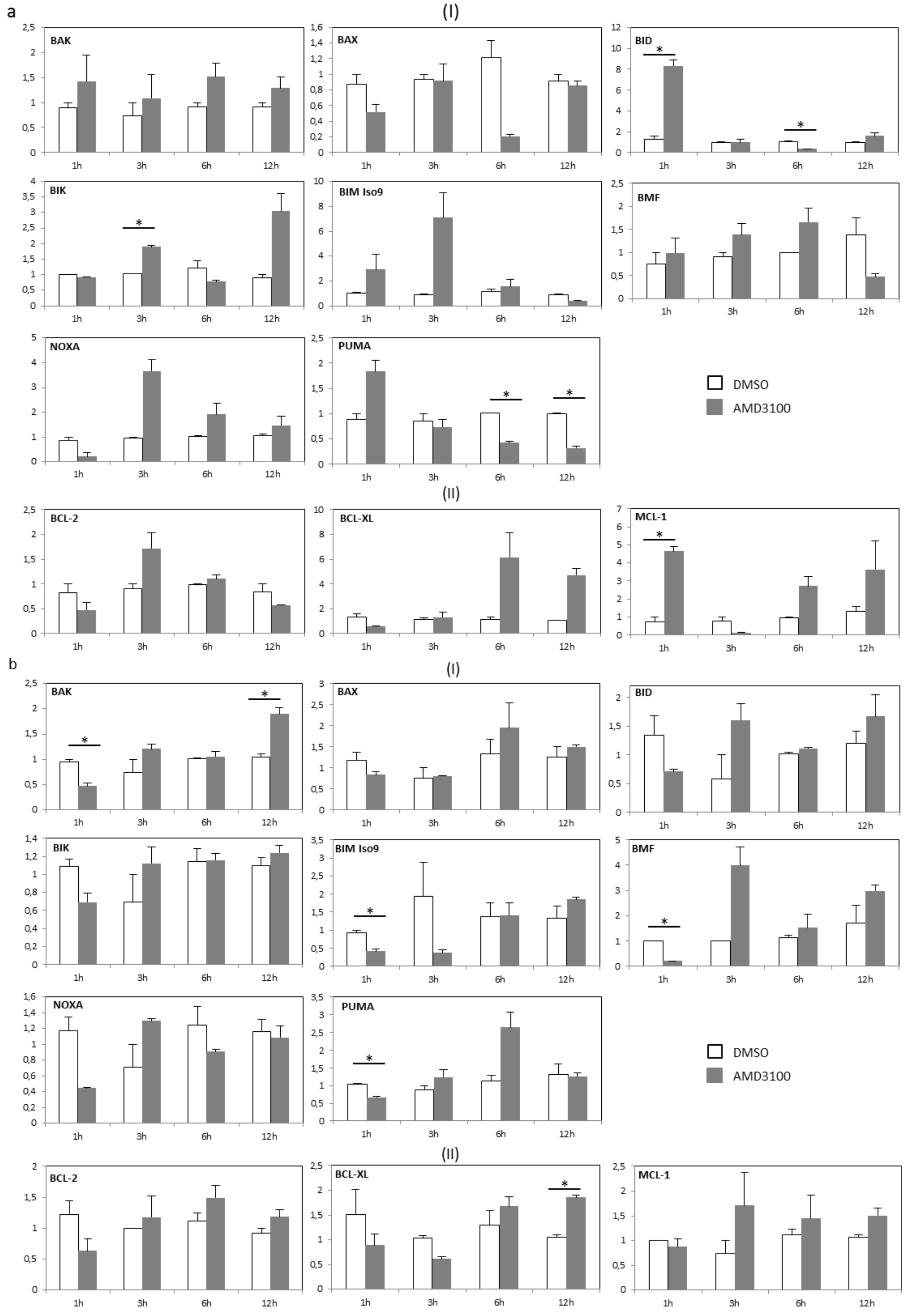

Figure S8. Gene expression of pro- and anti-apoptotic BCL2 members upon treatment with AMD3100. (**a**) BL2 and (**b**) U2932 cells were treated with 40 µM AMD3100 and after 1 h, 3 h, 6 h, and 12 h gene expression levels of (I) pro-apoptotic and (II) anti-apoptotic BCL2 members were determined by RQ PCR. mRNA expression levels

were calculated as the relative expression in comparison to DMSO treated controls. Assays were carried out in duplicate. Each bar represents the mean values of expression levels ± standard error of the mean (SEM). The comparison of the expression levels was performed by using the Mann–Whitney U-test or Student's t-test. * Expression is significant to the DMSO control ($p \leq 0.05$).

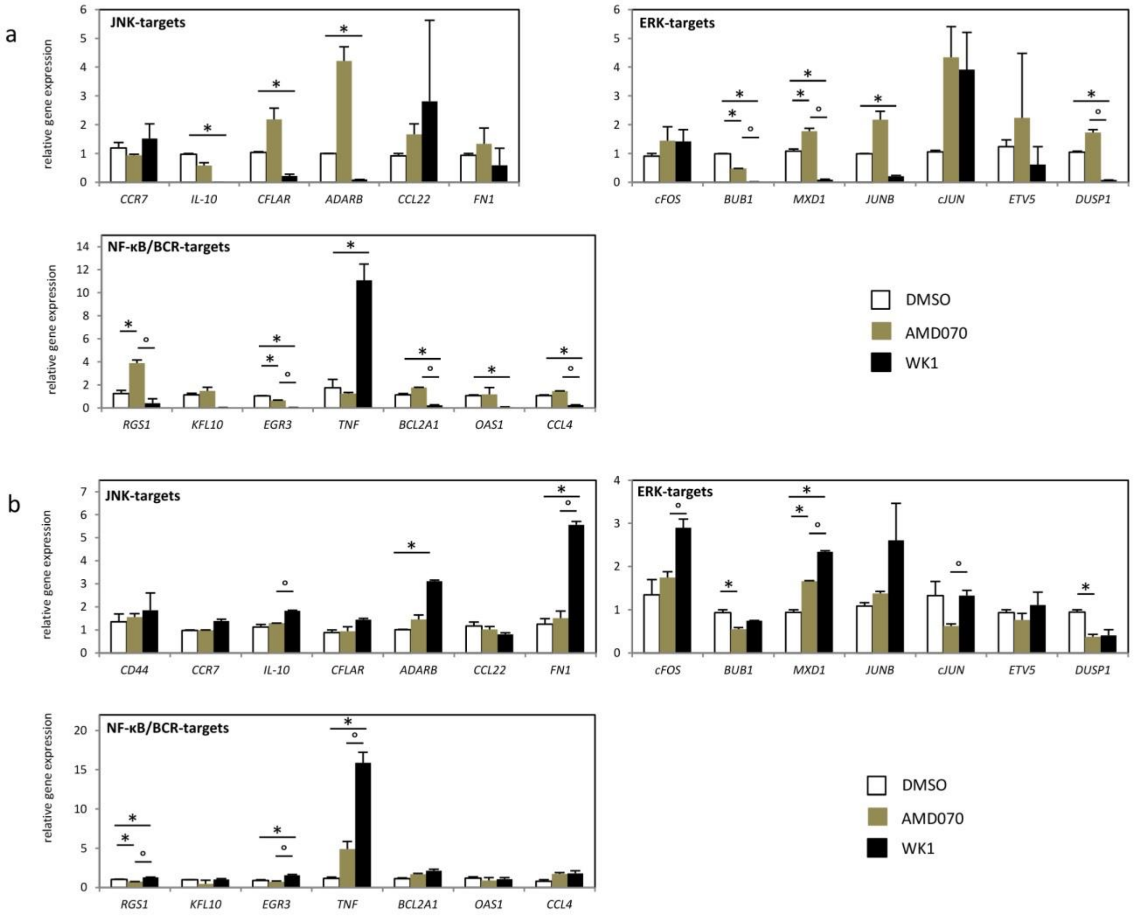

Figure S9: Gene expression of JNK-, ERK1/2-, and NF-κB/ BCR-target genes upon treatment with AMD070 and WK1. (**a**) BL2 (as Burkitt model) and (**b**) U2932 (as NGCB-DLBCL model) were treated with 40μM AMD070 or its niacin derivative WK1 and after 24 h gene expression levels of CCR7, IL-10, CFLAR, ADARB, CCL22, and FN as JNK-targets; cFOS, BUB1, MXD1, JUNB, cJUN, ETV5 and DUSP1 as ERK1/2; and RGS1, KLF10, TNF, BCL2A1, OAS1, and CCL4 as NF-κB/ BCR targets were determined in comparison to the DMSO treated control cells by RQ PCR. mRNA expression levels were calculated as the relative expression in comparison to the DMSO controls. Each bar represents the mean values of the expression levels ± standard error of the mean (SEM). The comparison of the expression levels was performed by using the Mann–Whitney U-test or Student's t-test. * Expression is significant to the DMSO control ($p \leq 0.05$). ° Expression is significantly different when comparing the AMD070 and WK1 treatment ($p \leq 0,05$).

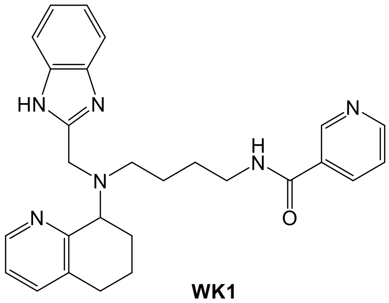

WK1

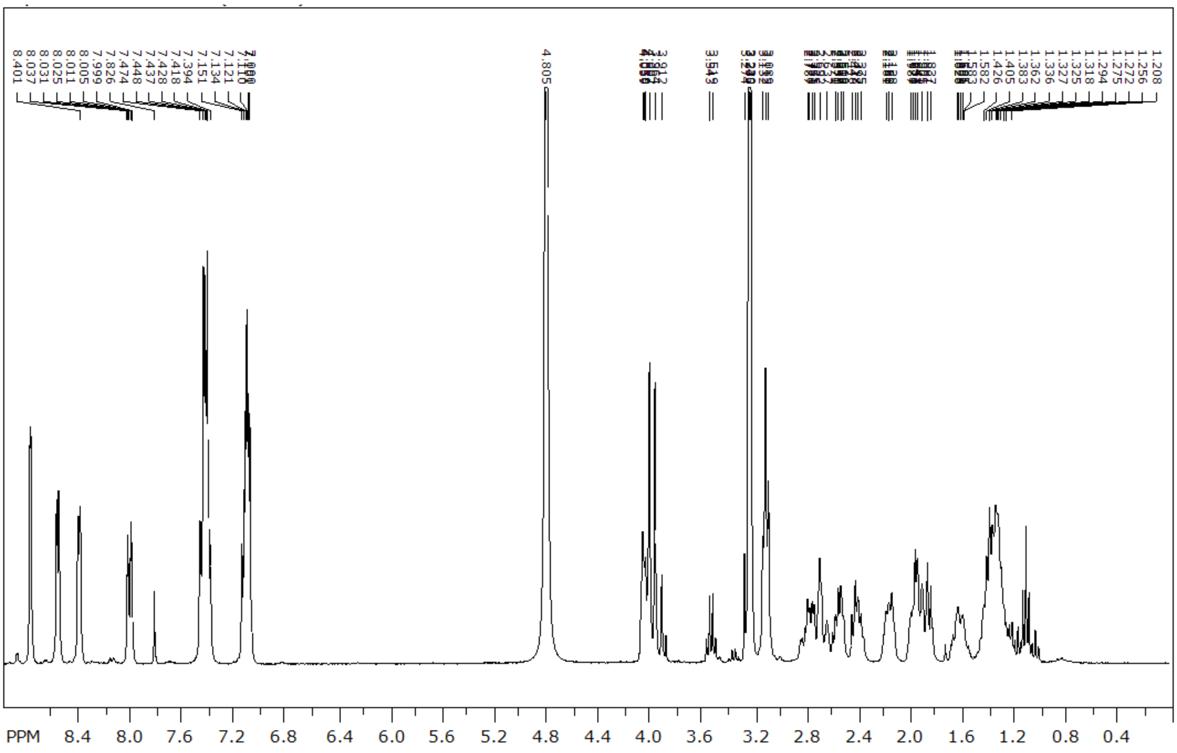

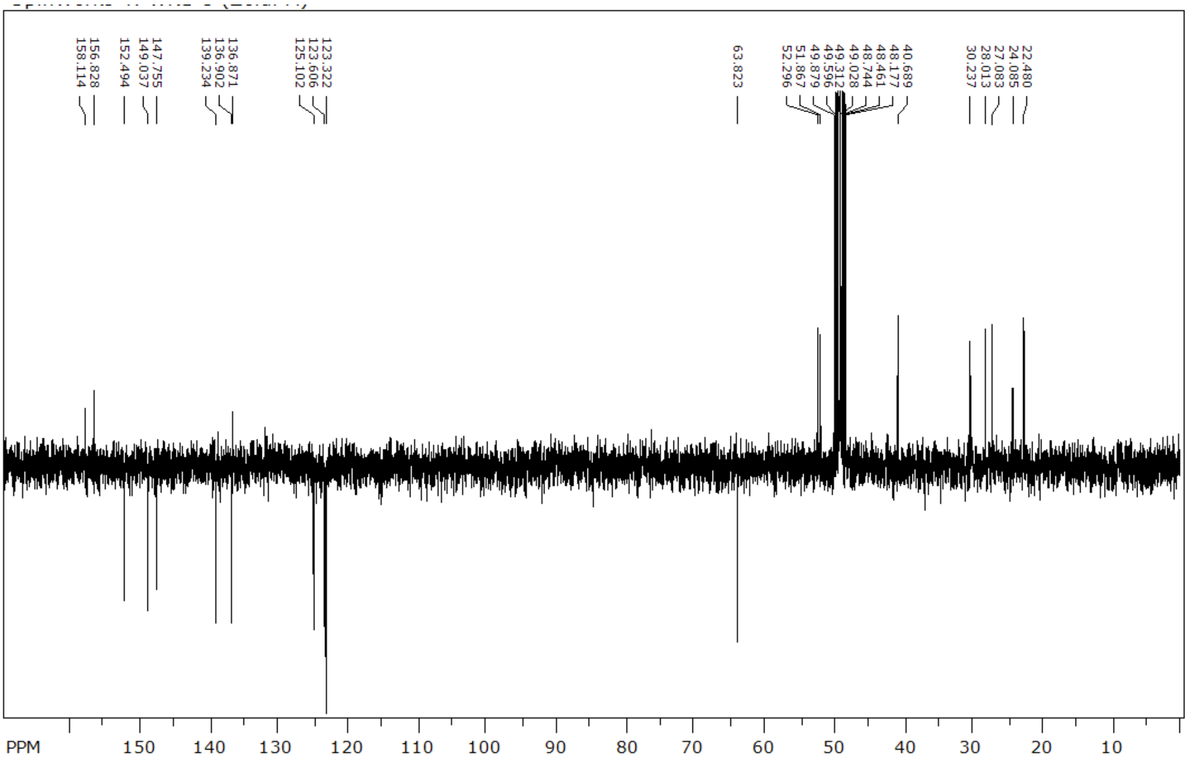

Figure S10. Chemical structure and nuclear magnetic resonance (NMR) spectra of WK1.

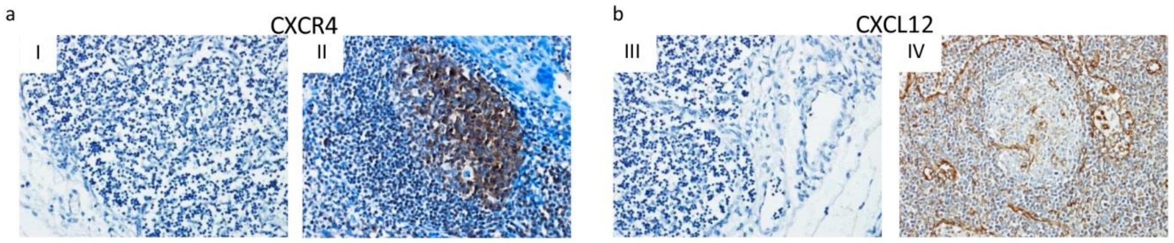

Figure S11. Negative (I & III) and positive (II & IV) controls for (**a**) CXCR4 and (**b**) CXCR7. Reactive tonsils were used for testing the two antibodies. All images (magnification 20×) were captured using an Olympus BX51 microscope and an Olympus E-330 camera**.**

Table S1. BCL2 status of the used lymphoma cell lines with type of DLBCL and references.

**Lymphoma cell lines**	**Disease**	**BCL2 status**	**References**	
SUDHL4	GCB-DLBCL	BCL-2-positive; major rearrangement in the BCL-2 gene	Klanova et al. 2015 Clinical Cancer Research [1]	Masir et al. 2010 Pathology [2]
RI-1	NGCB-DLBCL	BCL-2-positive; amplification of BCL-2	Klanova et al. 2015 Clinical Cancer Research [1]	Masir et al. 2010 Pathology [2]
U2932	NCGB-DLBCL	BCL-2-positive; overexpress BCL-2	Amini et al. 2002 Leukemia & Lymphoma [3]	Klanova et al. 2015 Clinical Cancer Research [1]
BL-2	Burkitt's Lymphoma	express BCL-2	Finke et al. 1992 Blood [4]	
Raji	Burkitt's Lymphoma	express BCL-2	Finke et al. 1992 Blood [4]	

Table S2. Oligonucleotide sequences of used primers.

GAPDH3428-f	AAG GTC GGA GTC AAC GGA TTT
GAPDH3428-r	ACC AGA GTT AAA AGC AGC CCT G
HPRT1-f	ATG GGA GGC CAT CAC ATT
HPRT1-r	ATG TAA TCC AGC AGG TCA GCA A
PPIA-f	CTC CTT TGA GCT GTT TGC AG
PPIA-r	CAC CAC ATG CTT GCC ATC C
CCL22	Qiagen #QT00089817
CCR7	Qiagen #QT01666686
CD44	Qiagen #QT00073549
IL10	Qiagen #QT00041685
MMP2	Qiagen #QT00088396
FN1	Qiagen #QT00038024
COL1A	Qiagen #QT00037793
CFLAR	Qiagen #QT00064554
ADARB	Qiagen #QT00081655
EGR3	Qiagen #QT00246498
cFOS	Qiagen #QT00007070
BUB1	Qiagen #QT00082929
MXD1	Qiagen #QT00082915
JUNB	Qiagen #QT00201341
cJUN	Qiagen #QT00242956
ETV5	Qiagen #QT00009485
DUSP1	Qiagen #QT00036638
CCL3-f	CTC CAA GCC CGG TGT CAT CT
CCL3-r	TTC TGG ACC CAC TCC TCA CT GG
CCL4-f	TAT GAG ACC AGC AGC CTC TG
CCL4-r	GCT TCT TTT GGT TTG GAA TAC C
KLF10-f	ATG CTC AAC TTC GGT GCC T
KLF10-r	TTC CAT TCT TTC CTC CGC
OAS3-f	CTG GTG TCC ACA GCC CTG AA
OAS3-r	TGC CAG AAC TGA GCT GCC C
RGS1-f	AAC TTC TTG CCA ACC AAA CTG
RGS1-r	CAA GCC AGC CAG AAC TCA
TNF-f	AAG CCT GTA GCC CAT GTT GTA G
TNF-r	AGA TGA GGT ACA GGC CCT CTG A
BCL2A1-f	CAC AGG AGA ATG GAT AAG GCA A
BCL2A1-r	TGA TTG TGC CAT TTC CCC C
BAD-f	GGT AGG AGC TGT GGC GAC T
BAD-r	CAA GCA TCA TCG CCA GG
PUMA-f	CGG AGA CAA GAG GAG CA
PUMA-r	ATG ATG AGA TTG TAC AGG ACC
BAX-f	CCT TTT CTA CTT TGC CAG CAA AC
BAX-r	GAG GCC GTC CCA ACC AC
BCL-XL-f	CAG TGA CCT GAC ATC CCA GC
BCL-XL-r	CCC ATA GAG TTC CAC AAA AGT ATC C
BCL-2-f	GGA GGA TTG TGG CCT TCT TTG
BCL-2-r	GCC GGT TCA GGT ACT CAG TCA T
MCL-1-f	CCA AGG ACA CAA AGC CAA TG
MCL-1-r	AAG AAC TCC ACA AAC CCA TCC
BIK-f	CTT GAT GGA GAC CCT CCT GTA TG
BIK-r	AGG GTC CAG GTC CTC TTC AGA
BAK-f	ATGGTCACCTTACCTCTGCAA
BAK-r	TCATAGCGTCGGTTGATGTCG
BIM Isoform 9-f	AAC CAC TAT CTC AGT GCA ATG G

BIM Isoform 9-r	TTG ACT ATG GTG GTG GCC A
BID-f	GGA ACC GTT GTT GAC CTC AC
BID-r	GAG GAG CAC AGT GCG GAT
BMF-f	TTC AAA GCA AGG TTG TGC AG
BMF-r	TTG TGG GGT GAC TGA GGA AC
NOXA-f	AGC TGG AAG TCG AGT GTG CT
NOXA-r	TCC TGA GCA GAA GAG TTT GGA
CXCR4	Qiagen #QT00223188
CXCR7	Qiagen #QT00069650
CXCL12	Qiagen #QT01008133
CXCR4-ex1 seq fw	CAGCAGGTAGCAAAGTGAC
CXCR4-ex1 seq rev	TCAAGAAAACTCCTTTCGGTG
CXCR4-ex2 seq fw	ATGGGAAAAGATGGGGAGG
CXCR4-ex2 seq rev	AGACTCAGACTCAGTGGAAAC

References

1. Klanova, M.; Andera, L.; Brazina, J.; Svadlenka, J.; Benesova, S.; Soukup, J.; Prukova, D.; Vejmelkova, D.; Jaksa, R.; Helman, K.; et al. Targeting of BCL2 Family Proteins with ABT-199 and Homoharringtonine Reveals BCL2- and MCL1-Dependent Subgroups of Diffuse Large B-Cell Lymphoma. *Clin. Cancer Res.* **2016**, *22*, 1138–1149, doi:10.1158/1078-0432.CCR-15-1191.
2. Masir, N.; Campbell, L.J.; Jones, M.; Mason, D.Y. Pseudonegative BCL2 protein expression in a t(14;18) translocation positive lymphoma cell line: a need for an alternative BCL2 antibody. *Pathology* **2010**, *42*, 212–216, doi:10.3109/00313021003631296.
3. Amini, R.-M.; Berglund, M.; Rosenquist, R.; Heideman, A. von; Lagercrantz, S.; Thunberg, U.; Bergh, J.; Sundström, C.; Glimelius, B.; Enblad, G. A Novel B-cell Line (U-2932) Established from a Patient with Diffuse Large B-cell Lymphoma Following Hodgkin Lymphoma. *Leuk. Lymphoma* **2002**, *43*, 2179–2189, doi:10.1080/1042819021000032917.
4. Finke, J.; Fritzen, R.; Ternes, P.; Trivedi, P.; Bross, K.J.; Lange, W.; Mertelsmann, R.; Dolken, G. Expression of bcl-2 in Burkitt's lymphoma cell lines: induction by latent Epstein-Barr virus genes. *Blood* **1992**, *80*, 459.
